# Ghrelin and Functional Dyspepsia

**DOI:** 10.1155/2010/548457

**Published:** 2010-01-12

**Authors:** Takashi Akamizu, Hiroshi Iwakura, Hiroyuki Ariyasu, Kenji Kangawa

**Affiliations:** ^1^Ghrelin Research Project, Translational Research Center, Kyoto University Hospital, Kyoto University School of Medicine, 54 Shogoin-Kawaharacho, Sakyo-ku, Kyoto 606-8507, Japan; ^2^National Cardiovascular Center Research Institute, Osaka 565-8565, Japan

## Abstract

The majority of patients with dyspepsia have no identifiable cause of their disease, leading to a diagnosis of functional dyspepsia (FD). While a number of different factors affect gut activity, components of the nervous and endocrine systems are essential for normal gut function. Communication between the brain and gut occurs via direct neural connections or endocrine signaling events. Ghrelin, a peptide produced by the stomach, affects gastric motility/emptying and secretion, suggesting it may play a pathophysiological role in FD. It is also possible that the functional abnormalities in FD may affect ghrelin production in the stomach. Plasma ghrelin levels are reported to be altered in FD, correlating with FD symptom score. Furthermore, some patients with FD suffer from anorexia with body-weight loss. As ghrelin increases gastric emptying and promotes feeding, ghrelin therapy may be a new approach to the treatment of FD.

## 1. Introduction

Dyspepsia is associated with a variety of organic and functional disorders. The organic causes of dyspeptic symptoms include peptic ulcers, cholelithiasis, reflux disease, and malignancy. In patients initially presenting with dyspepsia, approximately 33% to 50% have an underlying organic disease [1]. Routine clinical evaluation and procedures do not reveal the cause of symptoms in the majority of patients with dyspepsia. If symptoms persist for more than three months with symptom onset of at least six months prior to the diagnosis, affected patients are diagnosed with functional dyspepsia (FD). 

Despite an absence of organic disease in patients with FD, abnormalities in gastric emptying, dysregulation of gastroduodenal motility, and visceral hypersensitivity are often associated with FD. As ghrelin affects gastric motility/emptying [2–7] and secretion [8, 9], this peptide may play a pathophysiological role in FD. It is also possible that the functional abnormalities in FD may affect the production of ghrelin by the stomach. Indeed, plasma ghrelin levels are reported to be altered in FD [10, 11], frequently correlating with FD symptom score [11, 12]. Some patients with FD also suffer from anorexia with significant weight loss, frequently leading to diagnosis of eating disorders. As yet, no treatment guidelines for patients with FD or eating disorders have been established. Given its role triggering eating behaviors, ghrelin might be an appropriate treatment for FD, potentially improving food intake by influencing gastric emptying and motility. In this article, we discuss FD and the potential role of ghrelin in this disease.

## 2. Functional Dyspepsia

Functional dyspepsia (FD), a functional gastroduodenal disorder (FGDD) [13, 14] and one of the functional gastrointestinal disorders (FGIDs) [15], was previously known as nonulcer dyspepsia (NUD), essential dyspepsia, or idiopathic dyspepsia [16, 17]. The original Rome I criteria, which established the classification and diagnosis of FD in 1994 [18], have since been updated twice as Rome II in 2000 [19] and Rome III in 2006 [15]. 

The Rome II (Table 1) [19] defines FD as persistent or recurrent pain or discomfort centered in the upper abdomen. Discomfort refers to a subjective, negative feeling characterized by or associated with non-painful symptoms such as upper abdominal fullness, early satiety, bloating, or nausea. For a diagnosis, symptoms must have persisted or recurred for at least 12 weeks over a 12-month period. A dyspepsia subgroup classification was also proposed for research purposes, based on the predominant (most bothersome) symptom, (a) ulcer-like dyspepsia features pain (from mild to severe) as the predominant symptom, while (b) dysmotility-like dyspepsia exhibits discomfort (not pain) as the predominant symptom. 

The Rome III system radically reformulated the classification of FD (Table 2) [14]. The description of symptoms centered in the upper abdominal area was refined by the Committee to pain in the precise epigastric region, while other key symptoms (early satiety and fullness) must be meal-related. According to Rome III, discomfort was abandoned as a key concept, replaced by post-prandial fullness and early satiety. The Rome III criteria moved away from subcategorization using the predominant symptom (dysmotility-like and ulcer-like dyspepsia of Rome II), although similar entities, (a) postprandial distress syndrome and (b) epigastric pain syndrome, are subsumed under the FD section (Table 3). The Committee recognized that there are no one symptoms present in the majority of patients previously labeled with a diagnosis of FD. The time course for diagnosis was made less restrictive for all functional disorders; diagnosis was acceptable for symptoms originating six, not 12, months prior to diagnosis when currently active (i.e., meet criteria) for at least three months [13]. For research purposes, the term FD was abandoned in favor of a new classification system described in Table 3. 

Although the etiology of FD remains unclear, a number of factors may play a role in the development of symptoms [1] (Table 4). Visceral hypersensitivity is thought to be critical in causing FD by enhancing perception and processing of gastrointestinal neural inputs [20]. In a study of experimentally induced gut distention, the majority of patients with FD developed greater discomfort than matched healthy controls. As increased gut sensitivity may affect gut motility, acid secretion, and gastric distension, hypersensitivity may underlie many of the symptoms of FD. Patients with FD often have concomitant GI motility disorders and psychiatric illnesses. Whether these disorders are an epiphenomenon or related to the underlying disease remains unclear. Symptom development and exacerbation of FD are often linked to stressful life events, causing the patient to seek medical help at that time. Thus, patients seeking care for FD symptoms are more likely to have active life stressors than those who remain undiagnosed, leading to bias in the sample population. External stressors may also affect intestinal function, which in connection with visceral hypersensitivity, may lead to increased symptom perception.

## 3. Relationship of Ghrelin to the GI Tract and Feeding Behaviors

Neural and hormonal communication between the gut and the brain modulate appetite, feeding, and digestion [21, 22]. In the integrated gut-brain-energy axis, gastrointestinal (GI) motility, gastric acid secretion, digestion, and defecation are coordinated with appetite, satiation, and metabolism. Both organic and functional GI disorders are associated with alterations of the physiologic factors regulating the gut-brain-energy axis. 

Multiple hormones are secreted by the gut and adipose tissues during feeding, digestion, and fasting, each of which can profoundly affect the GI tract [22]. A subset of these hormones directly affects secretory function along the GI tract (e.g., gastrin stimulates acid secretion by parietal cells); major sites of hormone action also include enteric and vagal neurons, the area postrema (AP) within the medulla, and the hypothalamic arcuate nucleus (HAN). The AP and the median eminence, which has a close relationship with the HAN, have greatly reduced or absent blood-brain barriers. Impulses from the AP travel along nerve projections to the adjacent nucleus tractus solitarius (NTS) and higher regions of the brain, while those from the HAN do to other parts of the hypothalamus. Several hormones can cross the blood-brain barrier, including those with specific transporters such as leptin, insulin, and ghrelin [23]. Hormones released from the gut act on both the enteric nervous system (ENS) to contribute to the migrating motor complex (MMC) cycle and the CNS to promote the gradual re-establishment of appetite. The plasma concentrations of leptin and glucagon-like peptide 1 (GLP1) are low during fasting, while those of ghrelin and orexin are high. Significant research has focused on hormones whose plasma concentrations increase during fasting, as it is hypothesized that these hormones strongly affect hunger and energy expenditure. In addition to systemic effects, fasting-associated hormones also strongly affect a myriad of GI functions, possibly preparing the GI tract for food reception. Such a role has been proposed for ghrelin, the first orexigenic hormone identified that is produced in and released from the stomach. Ghrelin has a well-established role in increasing appetite and food intake [24, 25] and in stimulating gastric emptying and acid secretion [26, 27]; these functions are mediated, at least in-part, via vagal nerve pathways [9, 28]. The details concerning relationship of ghrelin to the GI tract function and food intake are also described in several manuscripts of this special issue (see, e.g., “Ghrelin's effect on hypothalamic neurons”, “Ghrelin-NPY axis”, “Gastric ghrelin and leptin”, “Motilin and ghrelin in the dog,” and “Ghrelin and stress in GI tract”). 

## 4. Plasma Concentrations of Ghrelin in FD

The gut and brain are highly integrated, communicating bidirectionally through neural and hormonal pathways [29]. Psychosocial factors can significantly influence digestive function, symptom perception, disease presentation, and outcome. Functional GI disorders, in return, can alter the physiologic factors regulating digestion and feeding [22]. 

The circulating levels of hormones related to appetite regulation are altered in dyspeptic disorders. Patients with dysmotility-like dyspepsia have higher serum concentrations of leptin, which is associated with gastritis and *H. pylori *infection [30]. Given that leptin is produced within the stomach to activate vagal-nerve terminals, reduce appetite, and increase mucin secretion, leptin may have a protective role in the upper gut during states of injury [22]. Plasma ghrelin levels in patients with FD are, however, controversial. While the total ghrelin levels were reported to be significantly higher in patients with FD (32 dysmotility-like and 7 ulcer-like FD patients) [31], they were significantly lower in patients with dysmotility-like FD [10, 32]. Concerning the active ghrelin levels, they were decreased in patients with postprandial fullness and/or early satiation defined by the Rome III classification [33], whereas similar between dysmotility-like FD patients and healthy controls [10]. The reason for discrepancy in plasma ghrelin levels remains uncertain, warranting further investigation. 

In terms of pathogenetic implication of these alterations in plasma ghrelin levels, the relationship with gastric emptying time in these patients was investigated. Significant correlation between the active ghrelin levels and the *T*_max _ value [33] and delayed gastric empyting in the majority of patients with abnormally low total ghrelin levels [32] were observed. In addition, elevations in the acylated form of ghrelin (active ghrelin) were significantly associated with subjective symptom score in FD patients [12]. Increased ghrelin concentrations are also seen in patients with duodenal and gastric ulcers, suggesting a possible relationship to mucosal injury [34]. 

## 5. Ghrelin Administration to Patients with FD

The therapies currently available for the treatment of dyspepsia, and for FD specifically, target the underlying hypothesized pathophysiology, including increased gastric acid sensitivity, delayed gastric emptying, and *H. pylori* infection. Only a small proportion of patients, however, experiences symptomatic relief using these treatments [35]. New treatment modalities targeting impaired gastric accommodation, visceral hypersensitivity, and central nervous system dysfunction are currently under development. 

**Figure 1:**
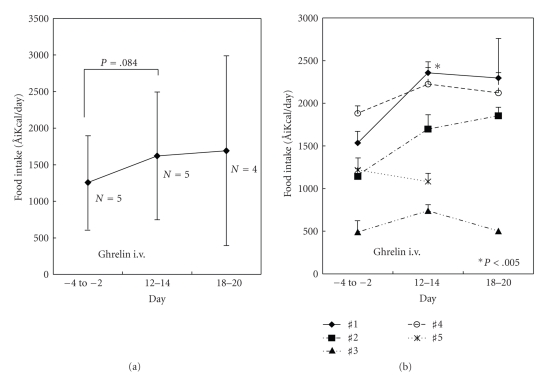
Daily food intake was measured before ghrelin injection (days −4 to −2), at the completion of treatment (days 12 to 14), and one week after injection (days 18 to 20) in five subjects who completed two weeks of ghrelin treatment. (a) Mean and 95% confidence interval (CI) for five subjects; (b) three-day means and SD of daily food intake for each subject.

In dysmotility-type FD, which comprises the largest subset of patients, abnormalities in gastrointestinal motility and sensitivity are thought to underlie the development of symptoms. Some patients with FD suffer from anorexia with weight loss, frequently leading to diagnosis with eating disorders. No treatment guidelines for patients with FD or eating disorders have been established. As ghrelin increases gastric emptying [2–6] and promotes feeding [36, 37], we investigated the ability of repeated ghrelin administrations to increase appetite and food intake in patients with FD [38]. We administered ghrelin by intravenous infusion (3 *μ*g/kg) twice a day before breakfast and dinner for two weeks to five patients. Ghrelin administration tended to increase daily food intake by approximately 30% in comparison to levels before and after completion of treatment, although this difference did not reach statistical significance (*P* = .084) (Figure 1(a)). Increases in food intake were maintained even one week after treatment (days 18–20). Although the precise mechanism is not known, the acute effects of ghrelin on gastric function may lead to sequential improvements in gastric mucosa and/or function. In addition, improvement in food intake may result from decreased anxiety or increased confidence concerning food intake in these patients. On an individual basis, food intake increased in four of the five subjects tested, decreasing in Patient #5 (Figure 1(b)). Food intake in Patient #1 was significantly elevated at the end of ghrelin treatment (days 12–14) from levels observed before treatment (*P* < .005). Hunger sensation was significantly elevated following a drip infusion (*P* < .0001). No severe adverse effects were observed. These results support the therapeutic potential of ghrelin in patients with FD. Additional studies, including larger placebo-controlled trials, will be necessary to confirm the usefulness of ghrelin in FD treatment.

## Figures and Tables

**Table 1:** Diagnostic criteria and classification of FD in Rome II.

Diagnostic criteria
At least 12 weeks, which need not be consecutive, in the preceding 12 months of
(1) Persistent or recurrent symptoms (pain or discomfort centered in the upper abdomen)
(2) No evidence of organic disease (including at upper endoscopy) that is likely to explain the symptoms
(3) No evidence that dyspepsia is exclusively relieved by defecation or associated with the onset of a change in stool frequency or stool form (i.e., not irritable bowel).

Classification

(B1a) Ulcer-like dyspepsia
Pain centered in the upper abdomen is the predominant (most bothersome) symptom.
(B1b) Dysmotility-like dyspepsia
An unpleasant or troublesome nonpainful sensation (discomfort) centered in the upper abdomen is the predominant symptom; this sensation may be characterized by or associated with upper abdominal fullness, early satiety, bloating, or nausea.
(B1c) Unspecified (nonspecific) dyspepsia
Symptomatic patients whose symptoms do not fulfill the criteria for ulcer-like or dysmotility-like dyspepsia.

**Table 2:** Dyspeptic symptoms defined by Rome III. This table is adopted by permission from Elsevier Limited [13, 15].

Symptom	Definition
Postprandial fullness	An unpleasant sensation akin to the prolonged persistence of food in the stomach

Early satiation	A feeling that the stomach is overfilled soon after starting to eat. This feeling is out of proportion to the size of the meal and results in the patient being unable to finish the meal

Epigastric pain	Pain located between the umbilicus and sternum in the midline of the torso. The pain is a subjective and unpleasant feeling, but difficult to describe. Some patients may describe feelings of tissue damage or chest pain

Epigastric burning	Pain located in the epigastrium that has a burning quality, but does not radiate to the chest

**Table 3:** Rome III criteria for functional gastroduodenal disorders. This table is adopted by permission from Elsevier Limited [13].

B			Functional gastroduodenal disorders
	B1		Functional dyspepsia (for application in clinical practice but not otherwise useful)
		B1a	Postprandial distress syndrome
		B1b	Epigastric pain syndrome
	B2		Belching disorders
		B2a	Aerophagia
		B2b	Unspecified excessive belching
	B3		Nausea and vomiting disorders
		B3a	Chronic idiopathic nausea
		B3b	Functional vomiting
		B3c	Cyclic vomiting syndrome
	B4		Rumination syndrome in adults

**Table 4:** Postulated mechanisms leading to the development of dyspeptic symptoms in patients with functional dyspepsia. This table is adopted by permission from Elsevier Limited [1].

Visceral hypersensitivity
(a) Increased perception of distention
(b) Impaired or altered perception of acid
(c) Visceral hypersensitivity secondary to chronic inflammation
Motility disorders
(a) Postprandial antral hypomotility
(b) Reduced relaxation of the gastric fundus
(c) Decreased or impaired gastric emptying
(d) Changes of the gastric electric rhythm
(e) Gastro-esophageal reflux
(f) Duodeno-gastric reflux
Changes in acid secretion
Hyperacidity
*Helicobacter pylori* infection
Stress
Psychological disorders and abnormalities
Genetic predisposition
